# Factors influencing utilization and perception of health care: a qualitative study among traumatized Yazidi refugees in Germany

**DOI:** 10.1186/s12888-021-03335-7

**Published:** 2021-07-12

**Authors:** Virginia M. Tran, Laila Fozouni, Jana K. Denkinger, Caroline Rometsch, Florian Junne, Patrick Vinck, Phuong Pham

**Affiliations:** 1 Department of Emergency Medicine, Highland Hospital-Alameda Health System, 1441 E 31st St, Oakland, CA 94602 USA; 2 Harvard Medical School, Boston MA, 25 Shattuck St, Boston, MA 02115 USA; 3 School of Medicine, University of California, San Francisco, 505 Parnassus Ave, San Francisco, CA 94143 USA; 4 Harvard Humanitarian Initiative, Harvard University, 14 Story Street, Cambridge, MA 02138 USA; 5 Medical University Hospital Tübingen, University of Tübingen, Osianderstraße 5, 72076 Tübingen, Germany; 6 Department of Psychosomatic Medicine and Psychotherapy, University Hospital, Otto von Guericke University Magdeburg, Magdeburg, Germany; 7 Harvard T.H. Chan School of Public Health, 677 Huntington Ave, Boston, MA 02115 USA; 8 Brigham and Women’s Hospital, 75 Francis St, Boston, MA 02115 USA

**Keywords:** Trauma, Refugees, Healthcare utilization

## Abstract

**Background:**

Ensuring adequate utilization of healthcare services for displaced populations is critical, yet there are well-documented treatment gaps. Yazidi women captured by the Islamic State (IS) were subjected to extreme trauma and violence. This study aims to understand perceptions of healthcare providers and utilization of these services among women who experienced extreme trauma.

**Methods:**

This is a qualitative study with voluntary participation offered to approximately 400 women resettled through the Special Quota Program. An empirical approach was used to collect data and a grounded theory approach was used for content analysis. Participants ranked their interactions with providers on a Likert scale. Posttraumatic stress disorder (PTSD) symptoms were assessed using the impact of event scale-revised questionnaire.

**Results:**

A total of 116 Yazidi women participated in this study. The women experienced an average of 6.8 months of captivity by IS and 93% met criteria for probable PTSD. Eighty-three percent of the women interacted with a physician; 80% found this interaction helpful. Sixty-nine percent interacted with psychologists; 61% found this interaction helpful. Six themes emerged: “reminders of trauma” and “hopelessness” in relation to the traumatic experience; “immediate relief” and “healing through pharmaceutical treatment” in relation to provider interventions, and “support” and “cultural differences” in relation to interactions with providers.

**Conclusions:**

There exist major barriers to care for Yazidi women who experienced extreme trauma, particularly in regards to psychiatric care. Perceptions of healthcare providers and perceived effectiveness of therapy are critical factors that must be taken into consideration to improve healthcare utilization and outcomes.

## Background

Refugees and other forcibly displaced populations frequently experience stressful events before, during, and after migration [1, 2] often leading to prolonged physical and mental suffering [3–7]. A high prevalence of mental illness such as post-traumatic stress disorder (PTSD) in these populations has been well documented [8–10]. The need for medical, psychiatric, and psychotherapeutic care among this population is recognized and has been associated with higher healthcare utilization in some settings [11, 12], but lower utilization of mental health care services elsewhere [13, 14]. Compared to local host populations, refugees have generally been found to have a large treatment gap, particularly in regards to psychiatric care [15, 16], and tend to have poorer health outcomes [17, 18].

Key barriers limiting healthcare access and utilization for refugees have been found to include availability of care, financial barriers, cultural and language barriers, and insufficient knowledge [19–22]. Avoidance behaviors associated with trauma-related disorders such as PTSD also appear to reduce entry into care, potentially perpetuating a cycle of lack of treatment, reduced resilience, continuation of PTSD symptoms, and further avoidance [23–26].

Despite the growing recognition of mental health care needs of refugees in a context of forced displacement [27], many studies on barriers to, utilization of, and satisfaction with psychiatric care focus primarily on the experiences of male veterans in western countries [28]. While some studies have assessed the experience of non-veteran populations with psychiatric care internationally [29], there remains a critical gap in understanding the experience of non-western female refugees. This study addresses this gap by examining qualitatively the experience of Yazidi women resettled in Germany.

Yazidis are a minority religious group from northern Iraq. Yazidis in the region of Sinjar were targeted for abduction, sexual and gender-based violence [30, 31], and ultimately genocide by fighters of the Islamic State (IS) [32]. In August 2014, IS attacked the Sinjar province of Iraq, where many Yazidis reside, committing massive human rights violations against the Yazidi community [33]. It has been estimated that in August 2014, 2.5% of the Yazidi population in Northern Iraq was either killed or kidnapped over the course of a few days [30]. Thousands of Yazidi women and girls were forced into sexual enslavement, and men were systematically murdered. Many experienced forced religious conversion, torture and sex slavery. Survivors witnessed the shooting, beheading, and live burning of their loved ones and community members [30]. The crimes committed by IS were later classified by the United Nations as genocide [33]. The population of traumatized Yazidi women have an estimated prevalence of post-traumatic stress disorder as high as 80% [34, 35].

This investigation aims specifically (1) to understand the perception of healthcare providers among Yazidi women who experienced severe and collective trauma and (2) to identify potential barriers to the women accessing and utilizing healthcare services in Germany. The qualitative design was selected to explore the rich and complex experience of women refugees dealing with psychiatric and medical care and to enable a thematic analysis that is increasingly valued in medical research [36, 37].

## Methods

This study was designed as an exploratory cross-sectional qualitative study of needs, utilization and barriers to psychiatric care among Yazidi women resettled in Germany. The protocol was approved by the Harvard Human Research Protection Program Institutional Review Board, Protocol # 17-0786, and by the ethics board of the University of Tübingen, Protocol # 348/2017BO1.

### Participants

Participants were selected among approximately 400 adult women resettled in Germany as part of the Special Quota Program (SQP). The SQP was established by the federal government of Baden-Württemberg, Germany, to provide assistance to traumatized Yazidi women who had experienced violence by IS and were internally displaced. The SQP assisted a total of 1100 Yazidis, of which 400 were adults and 700 were children. Resettlement took place in Germany beginning in 2015 with further assistance including housing, food, financial support, and access to healthcare. This level of assistance means that utilization of mental health care was not impeded by financial or knowledge barriers, providing a unique opportunity to isolate the impacts of trauma itself on access to healthcare and perception of healthcare providers.

Inclusion criteria included all SQP participants who were aged 18 or older at time of the interview. Approximately 400 of the 1100 SQP participants were aged 18 years or older when they arrived in Germany in 2015–16. The exact number was not available to the team due to privacy concerns. All eligible adult women Yazidi refugees in Germany assisted by the SQP were invited to participate in the study through their care network of social workers and accommodation center managers. A total of 116 adult women agreed to participate in the study out of approximately 400 eligible participants (29.0%). All 116 respondents had experienced the August 2014 ISIS attacks in Sinjar.

### Instrument and procedures

The questionnaire was developed by a team of epidemiologists, psychologists, and doctors from Harvard University and the Medical University Hospital of Tübingen. The instrument included both open-ended qualitative questions and structured questions assessing the participants’ overall experiences with professional healthcare assistance in Germany. Graphic representation of scales were utilized to facilitate selection of responses.

Questions were initially developed in English and German, then translated into the Kurmanji dialect spoken by the study participants. The interview guide was first piloted with 2 Yazidi/Kurdish women living in Germany to ensure cultural adequacy. Interviews were ultimately conducted by one female member of the study team from Harvard or Tübingen with a female interpreter. All interviewers and interpreters received several days of training prior to the start of data collection during which each question and the appropriate translation were discussed and agreed upon.

Recruitment materials included written documents outlining the purpose of the study and voluntary nature of participation. The material indicated that participation in the study would have no effect on the assistance provided by the SQP. The material also stressed the potential benefits and potential risks. No monetary compensation was offered.

Interviews were conducted between September 2017 and January 2018. Interviews lasted an average of 2 h and were conducted in a private setting by members of the study team and in the presence of an interpreter who spoke Kumanji. The interviews were audio-recorded in full, transcribed, and translated into English. The interview data and audio files were encrypted and kept on secured servers. Structured questions with 5-point Likert-scale and response options were directly inputted into KoboToolbox. All names and contact information were documented separately from all interview records.

Participants were asked about their interactions with healthcare professionals, including psychologists, psychotherapists, and medical physicians. Though the same questions were asked regarding the different types of healthcare professionals, the participants largely focused on their interactions with mental health professionals in their responses. Symptoms of PTSD were assessed using the impact of event scale-revised questionnaire (IES-R) [38]. A score of 33 or higher has been shown to be highly correlated with a clinical diagnosis of PTSD [39]. Basic demographic information were also collected.

### Data analysis

**Fig. 1:**
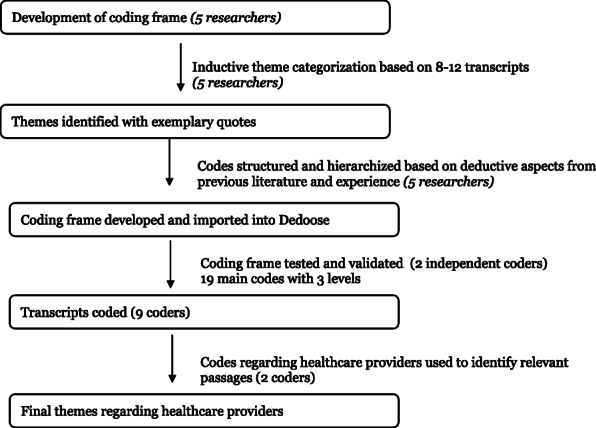
Qualitative Analyses and Coding Process

All interviews were professionally transcribed and translated. Data from transcripts were analyzed using qualitative content analysis according to Mayring approach [40], which is similar to other content analyses based on grounded theory (Fig. 1). First, a coding frame was developed by five interdisciplinary members of the research team. These team members conducted an initial thematic review of 8–12 transcripts each in order to inductively identify major categories and subthemes. In total, the team reviewed 50% of the total number of transcripts for the thematic review. These themes were discussed by the team and combined to yield a code tree with 19 main categories (root codes), 51 subcategories (parent codes), and 354 codes. Through structuring and hierarchizing the inductively derived codes based on deductive aspects from previous literature and the experience of the members of the research team, a coding frame was developed and imported into Dedoose (Version 8.0. 35). In a trial run, two independent coders tested the newly developed coding frame and compared their coding behavior. After finalizing the coding frame, with 19 main codes with 3 levels, and the definitions of the codes in another revision loop, the research team trained nine coders from Tuebingen and Boston. The coding team worked through the transcripts and assigned text passages to suitable codes, with one codeable text passage was defined as a passage that made sense by itself. In Dedoose, a count of code applications was used to observe the most commonly cited themes. This paper is a subset of a larger study for which these codes were created, and these codes were used to identify passages related to interaction with healthcare providers. Two independent researchers then identified the subset of themes described in this paper using the codes related to interactions with healthcare providers. At the final stage, themes were reviewed for coherence and consistency in relation to the coded text data. Frequencies and means were computed for sociodemographic variables and quantitative variables.

## Results

Between September 2017 and January 2018, 116 Yazidi adult women were interviewed for this study (Table 1).

**Table 1:** Characteristics of study participants

	Yazidi adult women (*n* = 116)
Age, mean (SD)	32.2 (8.2)
Marital Status	
*Married, n(%)*	68 (59%)
*Widow, n(%)*	15 (13%)
*Single, never married, n(%)*	32 (28%)
*Divorced, n(%)*	1 (1%%)
Location of partner if married (*n* = 68)	
*Iraq, n(%)*	24 (35%)
*Germany, n(%)*	17 (25%)
*Missing / unknown, n(%)*	24 (35%)
*Other country, n(%)*	3 (4%)
Literacy, n(%) literate	73 (63%)
Education level	
*None, n(%)*	33 (28%)
*Some primary, n(%)*	16 (14%)
*Finished primary, n(%)*	50 (43%)
*Higher than primary, n(%)*	17 (14%)
Employment status, n(%) employed	2 (2%)
Schooling status, n(%) studying	96 (83%)
Duration of presence in Germany, mean (SD) days	727.9 (90.0)
Duration of captivity by IS, mean (SD) months	6.8 (4.2)
Meet criteria for Post-Traumatic Stress Disorder, n(%)	101 (93%)
Interacted with doctors in Germany	
*Yes, n(%)*	96 (83%)
*No, n(%)*	10 (9%)
*No response, n(%)*	10 (9%)
Interacted with psychologists in Germany	
*Yes, n(%)*	80 (69%)
*No, n(%)*	30 (25%)
*No response, n(%)*	6 (5%)
Quality of interaction with doctors (*n* = 96)	
*Very unhelpful, n(%)*	10 (10%)
*Unhelpful, n(%)*	5 (5%)
*Neutral, n(%)*	4 (4%)
*Helpful, n(%)*	14 (15%)
*Very helpful, n(%)*	63 (66%)
Quality of interaction with psychologists (*n* = 80)	
*Very unhelpful, n(%)*	19 (24%)
*Unhelpful, n(%)*	4 (5%)
*Neutral, n(%)*	8 (10%)
*Helpful, n(%)*	7 (9%)
*Very helpful, n(%)*	42 (53%)

The women had, on average, experienced 6.8 months of captivity, and almost all (93%) met criteria for a possible diagnosis of PTSD [37]. Most had interacted with a medical doctor (83%) and/or a psychologist (69%). More Yazidi women found interacting with medical doctors helpful (81%) compared to the percentage that found their interaction with psychologists helpful (62%).

**Fig. 2:**
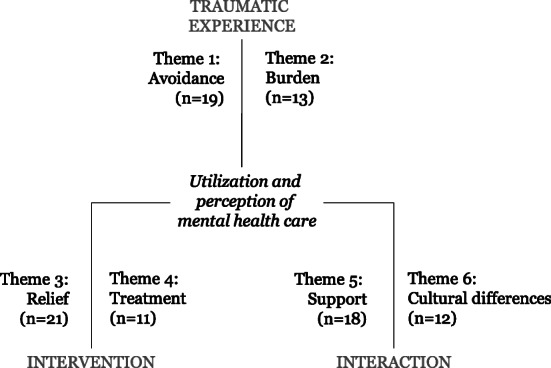
Thematic map of Yazidi perception of care

Participants reported six themes relating to their utilization and perception of care. For this analysis, the themes were structured in three groups: themes related to traumatic exposure (reminders of trauma, hopelessness), to intervention (immediate relief, healing through pharmaceutical treatment) and interaction (support, cultural barriers) (Fig. 2).

### Traumatic experience

Participants frequently mentioned aspects related to their traumatic experience as a barrier to receiving treatment, including reminders of past trauma in the context of taking part in care, as well as a sense of an insurmountable burden and hopelessness.

### Theme 1: reminders of trauma

Reminders of trauma were identified as an important theme, with respondents identifying the fear of having to retell and remember their traumatic history as a barrier (theme: reminders of trauma). Some participants found that speaking about their past triggered unwanted memories and worsened their psychological health. The women reported experiencing “violence” or distress after their interactions with providers.

*“It didn't help me [going to psychologist]. He returned me and I felt as if I experienced violence.” - Participant 59*

“*No, they make me remember more. Yes, when I’m with them, I remember even more.” - Participant 43*

Furthermore, some women reported experiencing somatic symptoms, such as headaches, when asked about their trauma history.

“*When the woman asked me questions I had a headache. I told the person responsible for me here that I wasn’t going to go anymore.” - Participant 102*

*“Now I have a headache when I talk about it. I don't want to experience any headache, I have a terrible headache now, because I talked about it.” - Participant 019*

Others experienced negative emotions including anger, anxiety, and sadness when asked by care providers to speak about their time in Iraq.

*“When we went to talk, we got even more upset.” - Participant 35*

They mentioned wanting to forget these memories in order to create new lives for themselves in Germany but found it harder to do so when asked to speak about their experiences. They reported worsened mental health from having to disclose their trauma. These reminders of past trauma caused participants to avoid taking part in care altogether.

“*The day I talk to the psychologist, everything rushes back to my mind and I can’t forget. I feel more troubled when it happens.” - Participant 2*

*“When you go and see a psychologist, they will ask you to tell your story from the beginning and that makes me sad and think again about what happened to me. In Iraq, I visited psychologists several times; they made my state even worse.” - Participant 98*

“*When I talked, I used to cry, I used to leave the group because it reminded me of what happened to me. I told them that I wanted to forget, and whenever I talk about it, I remember.” - Participant 81*

### Theme 2: hopelessness

Participants also expressed that the magnitude of their traumatic experiences was too great to overcome (theme: hopelessness). Some of them mentioned good, trusting relationships with their doctors, but still asserted that no amount of support or counseling would ever help them overcome the trauma they had experienced.

*“Doctors cannot cure what we experienced” - Participant 35*

*“We are hopeless case” - Participant 78*

*“The psychotherapist came here and I said no. I went twice to him but I don't feel like any human will be capable of healing me unless my heart feels fine.” - Participant 25*

*“They (the doctors) are good, when we have a problem, there are some doctors who you can trust, but you know they can’t do anything with man's heart or mind.” - Participant 115*

In addition, some felt that although providers are well-intentioned, the providers will never truly understand what the women had experienced and how much they had suffered (subtheme: understanding).

“*You know the countries like these they don't mind as much as us. They tell us to go disco, to go to pool, go I don't know where, we cannot. - We tell them our son, sisters, husband is lost, they say ok, is this what all the life is about?” - Participant 1*

*“I went to the Psychologist, they don't help. They don't understand us.” - Participant 1*

Many of the study participants witnessed the murders of their loved ones, and many had family members who remain missing or in IS captivity. Some women believed that the safety of their family members was an essential prerequisite to their ability to recover from their trauma and achieve happiness (sub-theme: resolution). In some cases, the women cited the impossibility of bringing back murdered loved ones as a barrier to their ability to take part in care and healing.

*“The people who come to us to talk, it doesn’t change anything for our psychology. We want the captives to be helped. We want them to be rescued from the hands of those evil- doers.” - Participant 53*

*“No, no one can help me with this...No, nobody can bring my loved ones back to me.” - Participant 41*

### Utilization and perception of mental health care: intervention

Two themes related to the care or intervention itself emerged from the interviews: the potential or realized relief resulting from mental health care and the availability of treatment.

### Theme 3: immediate relief

In contrast to the fear of retelling their story of captivity or hopelessness, many women who received treatments from their mental health and/or medical services expressed a sense of immediate relief upon speaking to their healthcare providers about their traumatic experiences (theme: immediate relief). These women felt comfortable sharing their experiences with providers, and found that talking about their experiences actually helped them overcome their traumas.

*“I pour my troubles out, and I get relieved. I tell them things that I can’t tell anyone else.” - Participant 49*

*“When I talk to them, I empty my heart and I feel calm and relaxed.” - Participant 101*

*“I feel my heart is full of pain, when I talk I feel like it’s bleeding the pains away and letting go, and the doctor gave me very good pieces of advice.” - Participant 87*

*“I go and talk and feel better in that way.” - Participant 63*

The immediate relief experienced by the women through their interactions with providers, however, was not always due to recounting their stories of trauma. For some women, their time with providers provided relief by serving as a distraction from their experiences (subtheme: distraction). The providers served as a source of wanted distraction for the women by creating positive activities for the women to participate in or focusing conversations on topics other than their trauma, thus stabilizing their mood.

*“He visits me once a week. He doesn’t ask questions. He is very good with us … we are four sometimes. We do some activities together. We make flowers and stuff … He is really good.” - Participant 98*

*“Well, we talk and it’s good for us. We forget our problems.” - Participant 47*

### Theme 4: healing through pharmaceutical treatment

Beyond short-term relief from talking about their experiences, participants identified the possibility of more sustained symptomatic relief from their pharmaceutical treatments (theme: healing through pharmaceutical treatment) as a positive factor influencing perception and utilization of care. Many women suffered from physical and emotional pain as a result of being in IS captivity. The women reported positive experiences with psychological medications that were prescribed to them, which helped them deal with depression, anxiety, and insomnia.

*“You gave me medicine to help me, I was really in a dark place but after seeing a therapist and taking medicine I have become better” - Participant 91*

*“They prescribe pills which make me better. I get free from my fear, and I can forget it for the day. During the day I think about those killed by ISIS, I remember the times I was there, I remember dreadful things like this, but I don’t see any nightmares, and I don’t feel afraid when I use the pills.” - Participant 102*

The women reported feeling more sound of mind while on psychiatric medications. The medications and interactions with healthcare professionals helped some women overcome suicidal ideation.

*“I am mentally well balanced, thanks to the medication. I was harming my children. When I was in Iraq, I took a knife and tried to kill myself several times. I was running out on the streets at night, but now I'm fine thanks to the medications I use here.” - Participant 34*

### Utilization and perception of mental health care: interaction

Two additional themes emerged relating to the interaction of participants with healthcare professionals, relating to the sense of support they receive but also fundamental barriers resulting from cultural differences.

### Theme 5: support

Although not every woman may have experienced or anticipated relief from treatment, participants noted the supportive nature of their relationships with caregivers as a factor facilitating utilization and improving perception of caregivers (theme: support). Women noted feeling supported and less alone when providers cared for their wellbeing. They spoke to how having support from their care providers helped them find strength they otherwise would not have realized when alone.

*“I see myself not alone as they treat me … I mean that many people are with me and they support me.” - Participant 20*

*“They helped me and my children to become better. They take us to doctors; they are always ready to help. It feels good when you know that there are some people who are ready to help you doing something that you can't do them alone.” -Participant 98*

*“By God, yes. They helped me a lot, they gave me strength. Because I was weak when I went there. I kept on going there for a year. They gave me a lot of strength.” - Participant 51*

*“Like how I can look after myself, how I would not fear anything, like, another one, how I can believe in myself.” -Participant 109*

These women appreciated the mental health professionals who did not focus exclusively on their past trauma but rather helped them believe in their own abilities. The women felt supported when providers provided the women with advice regarding how to cope with non-medical issues, such as how to build stronger relationships with their loved ones and how to build their new lives in Germany through work and education. Conversations with providers allowed the women to focus on the future and their new lives.

*“He gave me so much strength, so much encouragement. How I can continue my life, how I can build a relationship with my son. He was very helpful.” - Participant 51*

*“Sometimes one does not know her situation and others say to her ‘You are in such a state. Do this thing because it may help you; and avoid that thing...When I tell my problems to her, she, for example, show me a path. This helps me the most.” - Participant 113*

*“Because it feels like that person knows what I have been through and he knows what my treatment is...He advises me...He tells me to go and do sports, go to school, make yourself stronger, go out, be a good example for your children.” - Participant 24*

Generally, women who had positive experiences with healthcare professionals reported feeling supported, less alone, and strengthened by their sessions with health professionals.

### Theme 6: cultural barriers

Finally, participants identified important cultural differences as a significant barrier, including language (theme: cultural barriers). Language barriers were present throughout the care cycle, with participants noting their inability to communicate their needs to social workers. This resulted in difficulties in scheduling medical appointments, finding Kurdish interpreters, and comprehending treatment instructions, such as the scheduling of medications.

*“We go when we have pain. But I have an infection for 1 year, and I am going to the doctors here. They are taking me there without an interpreter. I don’t understand any word they say. They cannot help me. I tell them that I have an infection and they understand nothing.” - Participant 28*

*“I don’t understand them when they come to me to help me. I have a problem with the language. I don’t have enough language skills to tell those women who come here to help me what to do. Even if they are Yazidi, I can't tell them what to do the way I want.” - Participant 115*

*“We do not really understand each other. We need to bring interpreters with us. They do not understand us.” - Participant 35*

The women also cited cultural barriers and differences as hindering effective communication and treatment. They felt as though they had too many cultural differences with their providers to find the advice of their providers meaningful. In this regard, they also perceived that their providers were unable to understand them and their needs. Among cultural barriers, some women expressed an aversion to medications prescribed by providers. For these women, they perceived medications as harmful or unnecessary. Many also endorsed stigma against taking medications, viewing it as a sign of weakness and a failure to cope with difficulties with one’s own strength. Others believed that medications would cause them more problems.

*“He didn't talk. He gave me medicines, I don't want to take medicines of psychology. They are not good. So, I said if I get over it with my own strength it is better.” -Participant 11*

*“I don't sleep at night, and I want to go to a doctor but I'm afraid. I went once to a therapist, but I didn't go again, I'm afraid to attend therapy Sessions, because if I go they will give me pills and medications and I can't take them. you know what! the women here take the pills, and it affects them, and if they don't take it, they will face side effects, so I can't take them.” - Participant 38*

*“No. I feel bad but I can't take medicines, I'm afraid I will feel like mad” - Participant 41*

## Discussion

Yazidi women who were forced into IS captivity were subjected to extreme violence and trauma. As a result, rates of post-traumatic stress disorder are higher among this population [34, 35] than reported previously in other similar populations, and ensuring unobstructed access to treatment is critical to recovery. There exists a large gap in the literature in understanding specific barriers to treatment for female victims of trauma in non-combatant and non-western populations. Thus, this study is an important step in filing this gap. We aimed to understand the factors influencing healthcare utilization for Yazidi women with a history of trauma resettled in Germany under the Special Quota Program. The Special Quota Program provided a unique case study in which to examine this question, since all of the women had their basic needs of shelter, safety, and food met by the program. With these needs met, and with access to free health care, many commonly reported barriers such as lack of knowledge or financial ability were eliminated. In addition, some contextual and structural factors were also controlled for as all the participants were from the same geographic area, ethnic and religious affiliation, and experienced IS captivity. In this study, themes of “reminders of trauma” and “hopelessness” were identified as important aspects of the traumatic experience negatively impacting the willingness to engage in care. On the other hand, among women who did received treatments the themes of “immediate relief”, “healing through pharmaceutical treatment”, “support”, and “cultural barriers” emerged as important factors impacting the perception of healthcare providers, with the subtheme of “distraction.” This finding is an encouraging indication that addressing factors relating to reminders of trauma, hopelessness, and cultural barriers may help the women achieve relief through treatment. These themes emerged in regards to both medical physicians and mental health professionals, though comments were largely directed towards mental health professionals despite fewer women reporting interactions with mental health professionals compared to medical physicians.

A major barrier to treatment among women in our study was the reminders of trauma associated with engaging in care, causing them to avoid interactions with healthcare professionals. In our study, patients reported immense distress and even somatic symptoms from having to retell their stories. The act of engaging in care often resurfaced painful memories, and this stress led the women to avoid engaging in care altogether. This finding is consistent with findings from prior studies in the literature, including a study among veterans with PTSD which found that many felt as though they lacked “emotional readiness” for treatment, and that engaging in care would increase anxiety levels [39]. Many participants also reported experiencing somatic symptoms, a well-established comorbidity of PTSD [41]. Avoidance of trauma-related stimuli is a hallmark feature of post-traumatic stress disorder, for which 93% of women in our study potentially met criteria [23, 24]. Existing literature attributes avoidance behavior to misperceptions regarding the “safety” of the treatment environment [42]. As such, some strategies that have been identified to deal with avoidance behavior include media campaigns to emphasize that treatment provides a safe environment at an individual pace, as well as promotion of individualized, patient-centered care [24]. Furthermore, our study suggests that low-threshold, informative interventions which explain the process of psychotherapy, would be useful. For instance, patients should be informed early on that engaging in care does not always mean they have to talk about their traumatic experiences. A stepped care and patient-centered approach [43] is recommended, and therapists must keep in mind that some patients might feel fearful about talking about their traumatic experiences. A stabilizing, supporting phase, focusing on the development of coping strategies and engagement in positive activities, might be necessary for this subgroup.

However, our finding is particularly informative in the context of the Special Quota Project, which necessitated engagement of the women with healthcare providers who came on site. Due to the requirements of the Special Quota Project, the majority of the women who expressed a desire to avoid receiving treatment had already engaged in care or were actively engaged in care during the time of the survey: 83% of all the women had engaged in care with a physician, and 69% with a psychiatrist or psychologist. In other words, the women had already experienced the treatment environment and still perceived it as harmful. For most of the women who expressed an experience of resurfacing of negative emotions or memories associated with their captivity, their comments were primarily directed towards their interactions with mental health professionals presumably in a context of trauma-focused therapy. This is in direct contrast to the subset of women who reported experiencing immediate relief when discussing their traumatic experiences with providers. It is important to note that our study primarily focused on women who experienced trauma and that we did not specifically identify participants with a specific diagnosis of PTSD. Furthermore, the psychologists in the program did not start therapy on all the women; many women were still being evaluated regarding their therapy needs and when they were ready to receive formal treatments. However, our findings raise important questions regarding the pace and sequence of psychotherapeutic treatments which focus on the traumatic experiences for victims of extreme trauma. This is especially true given the benefit that some women perceived when receiving support from providers in a more resource oriented, stabilizing manner. Our findings also show that it is hard to generalize, even in a homogenous sample such as ours. For some it might bring relief to talk about the traumatic experiences, while for others, it does not. This underlines the need for patient-centered mental health offers and the need for providers to assess potential fears of sharing traumatic experiences in the first contact to be able to meet the individual needs of each patient. Future studies should aim to identify strategies for overcoming barriers to engaging in care and deepening the understanding of the timeline for engaging in trauma therapy with extremely traumatized populations. Identifying strategies to overcome cultural barriers, which emerged as an important theme in our study and has also been well documented in the literature, is another important step to reducing barriers for this population [19, 44–46].

Furthermore, perception of hopelessness surfaced as a major theme impacting willingness to engage in care. Many of these women expressed feelings of hopelessness for their ability to recover. Though we did not screen for depression in our study, feelings of hopelessness can be an indicator for depression, which is a frequent comorbidity associated with post-traumatic stress disorder. In a study exploring the role of hopelessness in relation to PTSD and suicidality, pre-treatment hopelessness was found to be a significant moderator of overall PTSD symptom severity and self-perceived likelihood of suicide attempts [47]. In our study, many women who perceived a high burden of trauma refused therapy and interactions with health professionals due to the belief that nothing could help them. As such, hopelessness appears intimately tied with beliefs of ineffectiveness of therapy. This finding also suggests that improved education regarding the effectiveness of treatment could help overcome hopelessness as a barrier.

However, some women experienced relief from the treatment environment, found benefit from treatments including medications, and felt supported by providers. Previous studies have well established the benefit of psychosocial interventions for refugees experiencing PTSD symptoms [48, 49]. Our study suggests that overcoming avoidance behaviors by addressing reminders of trauma, feelings of hopelessness, and cultural barriers may be important first steps to improve perception of healthcare providers and health care utilization. Further studies should aim to better understand contextual factors and individual differences that impact perceptions of healthcare providers, and how to overcome barriers to effective treatment.

This study has several limitations. First, there is a potential selection bias as the women were not randomly selected for participation. Because participation in the study was voluntary, the women who participated in our study may have differed in willingness to engage with healthcare providers compared to those who did not. Unfortunately, due to the sensitive nature of this study, we were unable to evaluate how those who participated in our study differed from those who did not. However, those who participated in the research are likely more willing to engage with healthcare providers than those who did not. Also, since enrollment materials were distributed through caregiver networks, women who underutilized their resources and were less connected to the caregiver network may have been underrepresented in the sample. An additional limitation of our study is that there was variation in frequency of mental health visits offered among the different settlement sites in Germany, as well as possible differences in behavior and treatment approach of mental health providers, which could impact perception of treatment. The variability in spoken and written Kurmanji language presents an additional challenge in communicating effectively with some of the participants. Furthermore, while the cross-cultural validity of the IES-R tool has never been tested among our study population, an Arabic version has been used in other Middle Eastern regions. In addition, the whole questionnaire and interview guide was piloted with Yazidi/Kurdish women and the interpreters worked to ensure participants understood the interview questions correctly and clarified any confusion. This process was done under the supervision of mental health professionals on the research team.

## Conclusions

Despite these limitations, our research has important implications regarding the care of traumatized populations. This study is one of the first to document Yazidi women’s perceptions of healthcare professionals and the efficacy of their interactions. Our hope is that the study results can help inform approaches to clinical encounters with this specific population and potentially other minority immigrant populations who are exposed to conflict and genocide related violence. Because the women in our study received services through the Special Quota Program, we were able to better assess their perceptions of health care resources in the context of their experienced trauma without traditional financial and knowledge barriers. The women’s responses largely focused on mental health providers, but there was a lower rate of utilization of mental health services than physician services, highlighting the particular need to focus attention towards barriers in accessing psychological care. Our study has identified the importance of assessing a patient’s perceived ability to disclose trauma information in order to address healthcare avoidance. Furthermore, efforts should be made to address ideas of hopelessness and perceived effectiveness of therapy. Addressing these barriers while providing support for patients, with conversations focused on understanding cultural, religious, and specific trauma experience may lead to developing more targeted treatment plans for the women. This in turn could lead to increased healthcare utilization and positive health outcomes, and eventually better integration in Germany. Future research should explore strategies for improving trauma-focused therapy in refugee populations including stepped care delivery [50], identify clinical predictors of patient perception and utilization of health care services, develop effective processes of establishing space for engagement, and determine the ideal timing and duration of treatment.

## Data Availability

The datasets used and/or analyzed during the current study are available from the corresponding author on reasonable request.

## Abbreviations

PTSD: Post-traumatic stress disorder
IS: Islamic State
SQP: Special Quota Program
IES-R: Impact of event scale-revised questionnaire
